# Supplementary material for: A mutant Escherichia coli that attaches peptidoglycan to lipopolysaccharide and displays cell wall on its surface
Source: eLife. 2014 Dec 31;3:e05334. doi: 10.7554/eLife.05334 (PMC4296511; doi:10.7554/eLife.05334)
Supplement: Supplementary file 1. — (A) Synthetic interactions between waaL15 and mutations affecting the elongasome due to limited lipid II availability. (B) Strains used in this study. (C) Plasmids used in this study. DOI: http://dx.doi.org/10.7554/eLife.05334.009 [file elife05334s001.docx]

**SUPPLEMENTAL MATERIAL (Tables A, B, C; Supplemental References)**

**Supplementary file 1A: Synthetic interactions between *waaL15* and mutations affecting the elongasome due to limited lipid II availability.**

| **Relevant genes^*^** | |  | | | **Cotransduction** | |
| --- | --- | --- | --- | --- | --- | --- |
| **Donor** | **Recipient** | | | **Selected Allele** | **Gene** | **Frequency** |
| *yhfT3084*::Tn*10* *mrcA*::*kan* | *waaL⁺* | | | *yhfT-3084*::Tn*10* | *mrcA*::*kan* | 85% |
| *yhfT3084*::Tn*10*  *mrcA*::*kan* | *waaL15* | | | *yhfT-3084*::Tn*10* | *mrcA*::*kan* | 89% |
| *sfsB203*::Tn*10* *lpoA*::*kan* | *waaL⁺* | | | *sfsB203*::Tn*10* | *lpoA*::*kan* | 29% |
| *sfsB203*::Tn*10* *lpoA*::*kan* | *waaL15* | | | *sfsB203*::Tn*10* | *lpoA*::*kan* | 28% |
| *zad*-*220*::Tn*10*  *mrcB*::*kan* | *waaL⁺* | | | *zad*-*220*::Tn*10* | *mrcB*::*kan* | 72% |
| *zad*-*220*::Tn*10 mrcB*::*kan* | *waaL15* | | | *zad*-*220*::Tn*10* | *mrcB*::*kan* | 13% |
| *zce-726*::Tn*10* *lpoB*::*kan* | *waaL⁺* | | | *zce-726*::Tn*10* | *lpoB*::*kan* | 77% |
| *zce-726*::Tn*10* *lpoB*::*kan* | *waaL15* | | | *zce-726*::Tn*10* | *lpoB*::*kan* | 5% |
|  |  | | |  |  |  |
| **pMurA**† |  | |  | |  |  |
| *zad*-*220*::Tn*10 mrcB*::*kan* | *waaL⁺* | | | *zad*-*220*::Tn*10* | *mrcB*::*kan* | 79% |
| *zad*-*220*::Tn*10 mrcB*::*kan* | *waaL15* | | | *zad*-*220*::Tn*10* | *mrcB*::*kan* | 77% |
| *zce-726*::Tn*10* *lpoB*::*kan* | *waaL⁺* | | | *zce-726*::Tn*10* | *lpoB*::*kan* | 75% |
| *zce-726*::Tn*10* *lpoB*::*kan* | *waaL15* | | | *zce-726*::Tn*10* | *lpoB*::*kan* | 70% |

***** *mrcA* encodes PBP1A which functions with LpoA in the divisome; *mrcB* encodes PBP1B which functions with LpoB in the elongasome. † Expression of *murA* was induced by supplementing growth media with 100 μM IPTG.

**Supplementary file 1B: Strains used in this study.**

| **Strain** | **Relevant Genotype** | **Reference** |
| --- | --- | --- |
| MC4100 | F^−^ *araD139* ^−^ (*arg*-*lac*)*U169* *rpsL150* *relA1* *flbB5301 deoC1 ptsF25 thi* | (Casadaban, 1976) |
| NR754 | MC4100 Ara^+^ | (Button *et al.*, 2007) |
| CAG12025 | F^−^ *araD139* ^−^ *rph-1 zad-220*::Tn*10* | (Singer *et al.*, 1989) |
| CAG12072 | F^−^ *araD139* ^−^ *rph-1* *sfsB203*::Tn*10* | (Singer *et al.*, 1989) |
| CAG12078 | F^−^ *araD139* ^−^ *rph-1 zce-726*::Tn*10* | (Singer *et al.*, 1989) |
| CAG18456 | F^−^ *araD139* ^−^ *rph-1* *yhfT3084*::Tn*10* | (Singer *et al.*, 1989) |
| MG617 | NR754 Δ*lptE2*::*kan* / p*lptE*^+^ | (Malojčić *et al.*, 2014) |
| MG1029 | NR754 Δ*lptE2*::*kan* / p*lptE613* | (Malojčić *et al.*, 2014) |
| MG1088 | NR754 Δ*lptE2*::*kan* *waaL15*/ p*lptE613* | This study |
| MG1167 | NR754 Δ*lptE2* *waaL15*/ p*lptE613* | This study |
| MG1180 | MG1167 Δ*cpsG*::*kan* | This study |
| MG1181 | MG1210 Δ*bamE*::*kan* | This study |
| MG1182 | MG1211 Δ*bamE*::*kan* | This study |
| MG1196 | MG1210 *bamB*::*kan* | This study |
| MG1197 | MG1211 *bamB*::*kan* | This study |
| MG1210 | NR754 *waaL*^+^ *tdh*::Tn*10* | This study |
| MG1211 | NR754 *waaL15* *tdh*::Tn*10* | This study |
| MG1214 | MG1167 Δ*cpsG*::*kan rff*::Tn*10* | This study |
| MG1234 | MG1167 *rff*::Tn*10*-66 | This study |
| MG1378 | MG1210 *ompC::*Tn*5*::*kan rcsC137* | This study |
| MG1379 | MG1211 *ompC*::Tn*5 rcsC137* | This study |
| MG1642 | NR754 *waaL^+^* | This study |
| MG1643 | NR754 *waaL15* | This study |
| MG1635 | CAG12025 Δ*mrcB*::*kan* | This study |
| MG1636 | CAG18456 Δ*mrcA*::*kan* | This study |
| MG1671 | CAG12072 Δ*lpoA*::*kan* | This study |
| MG1672 | CAG12078 Δ*lpoB*::*kan* | This study |

**Supplementary file 1C. Plasmids used in this study.**

| **Plasmid** | **Description** | **Reference** |
| --- | --- | --- |
| p*lptE* | *lptE* cloned into pBAD18, Amp^R^ | (Wu *et al.*, 2006) |
| pMurA | ASKA plasmid with cloned *murA*, Cam^R^ | (Kitagawa *et al.*, 2006) |

**SUPPLEMENTAL REFERENCES:**

Button, J.E., Silhavy, T.J., and Ruiz, N. (2007) A suppressor of cell death caused by the loss of σ^E^ downregulates extracytoplasmic stress responses and outer membrane vesicle production in *Escherichia coli*. *J Bacteriol* **189**: 1523–1530.

Casadaban, M.J. (1976) Transposition and fusion of the *lac* genes to selected promoters in *Escherichia coli* using bacteriophage lambda and Mu. *J Mol Biol* **104**: 541–555.

Kitagawa, M., Ara, T., Arifuzzaman, M., Ioka-Nakamichi, T., Inamoto, E., Toyonaga, H., and Mori, H. (2006) Complete set of ORF clones of *Escherichia coli* ASKA library (a complete set of *E. coli* K-12 ORF archive): unique resources for biological research. *DNA Res* **12**: 291–299.

Malojčić, G., Andres, D., Grabowicz, M., George, A.H., Ruiz, N., Silhavy, T.J., and Kahne, D. (2014) LptE binds to and alters the physical state of LPS to catalyze its assembly at the cell surface. *Proc Natl Acad Sci USA* **111**: 9467–9472.

Singer, M., Baker, T.A., Schnitzler, G., Deischel, S.M., Goel, M., Dove, W., *et al.* (1989) A collection of strains containing genetically linked alternating antibiotic resistance elements for genetic mapping of *Escherichia coli*. *Microbiol Rev* **53**: 1–24.

Wu, T., McCandlish, A.C., Gronenberg, L.S., Chng, S.-S., Silhavy, T.J., and Kahne, D. (2006) Identification of a protein complex that assembles lipopolysaccharide in the outer membrane of *Escherichia coli*. *Proc Natl Acad Sci USA* **103**: 11754–11759.
